# Human Milk-Derived *Enterococcus faecalis* HM20: A Potential Alternative Agent of Antimicrobial Effect against Methicillin-Resistant *Staphylococcus aureus* (MRSA)

**DOI:** 10.3390/microorganisms12020306

**Published:** 2024-01-31

**Authors:** Eun-Ji Yi, Trang Thi Minh Nguyen, Xiangji Jin, Arce Defeo Bellere, Mi-Ju Kim, Tae-Hoo Yi

**Affiliations:** 1 Graduate School of Biotechnology, Kyung Hee University, Yongin 17104, Republic of Korea; 0201@khu.ac.kr (E.-J.Y.); trangnguyen@khu.ac.kr (T.T.M.N.); arcedbellere@khu.ac.kr (A.D.B.); 2 Department of Dermatology, School of Medicine, Graduate School, Kyung Hee University, 26 Kyungheedae-ro, Dong-daemun, Seoul 02447, Republic of Korea; hyanghe112@khu.ac.kr; 3 Department of Food Science and Biotechnology, Kyung Hee University, Yongin 17104, Republic of Korea

**Keywords:** methicillin-resistant *Staphylococcus aureus*, skin, antibiotic resistance, human milk, lactic acid bacteria, *Enterococcus faecalis*

## Abstract

The increasing global impact of skin diseases, fueled by methicillin-resistant *Staphylococcus aureus* (MRSA), emphasizes the necessity for alternative therapies with lower toxicity, such as lactic acid bacteria (LAB). This study aims to isolate potential LAB from human milk and evaluate their efficacy against MRSA using various methods, including well diffusion, microdilution, crystal violet assay, enzymatic characterization, SDS-PAGE, and scanning electron microscopy (SEM). Among the 26 LAB screened, the human milk-derived strain HM20 exhibited significant antimicrobial activity against *S. aureus* CCARM 3089 (MRSA), which is a highly resistant skin pathogen. Through 16S rRNA sequencing, strain HM20 was identified as closely related to *Enterococcus faecalis* ATCC 19433^T^, which was subsequently designated as *Enterococcus faecalis* HM20. The minimum inhibitory concentration (MIC) of the cell-free supernatant (CFS) of HM20 against *S. aureus* KCTC 3881 and *S. aureus* CCARM 3089 was determined to be 6.25% and 12.5%, respectively. Furthermore, the effective inhibition of biofilm formation in *S. aureus* KCTC 3881 and *S. aureus* CCARM 3089 was observed at concentrations of 12.5% and 25% or higher, respectively. The antibacterial effect of the CFS was attributed to the presence of organic acids, hydrogen peroxide, and bacteriocins. Additionally, the antimicrobial peptides produced by HM20 were found to be stable under heat treatment and analyzed to have a size below 5 kDa. SEM image observations confirmed that the CFS of HM20 caused damage to the cell wall, forming pores and wrinkles on *S. aureus* KCTC 3881 and *S. aureus* CCARM 3089. This comprehensive investigation on strain HM20 conducted in this study provides foundational data for potential developments in functional materials aimed at addressing skin infections and antibiotic-resistant strains in the future.

## 1. Introduction

The rise of antibiotic resistance, particularly in methicillin-resistant *Staphylococcus aureus* (MRSA), presents a significant challenge in healthcare settings and communities worldwide [1]. This bacterium has developed the ability to evade the innate and adaptive immune responses of the host. This leads to its ability to colonize human hosts and cause infections, primarily in the skin and soft tissue [2,3].

The human skin, the largest organ of the body, faces constant exposure to the external environment, making it both a vulnerable target and a crucial first line of defense against pathogens [4]. Generally, anything that is in contact with the skin can be a potential agent for the infection of *S. aureus*. The emergence of resistance to penicillin and methicillin, the β-lactam antibiotics historically used for staphylococcal infections, began in the 1950s, posing an ongoing challenge to treatment [5]. MRSA eradication is particularly difficult due to its persistence in household settings and its ability to evolve resistance to specific medications [6]. This resistance stems from its ability to manipulate the host’s adaptive immune responses, effectively evading nearly every aspect of the immune system [2,3]. Notably, Malachowa and DeLeo identified MRSA resistance to trimethoprim, erythromycin, clindamycin and tetracycline [7], which is acquired through resistance genes like *mecA* on the mobile genetic element cassette chromosome *mec* (SCC*mec*) [8,9]. Even vancomycin, the current treatment of choice, has faced growing reports of resistance due to polygenic mutations in the cell wall biosynthesis gene *vanA* and its associated operon located on plasmids [10,11].

**Figure 1:**
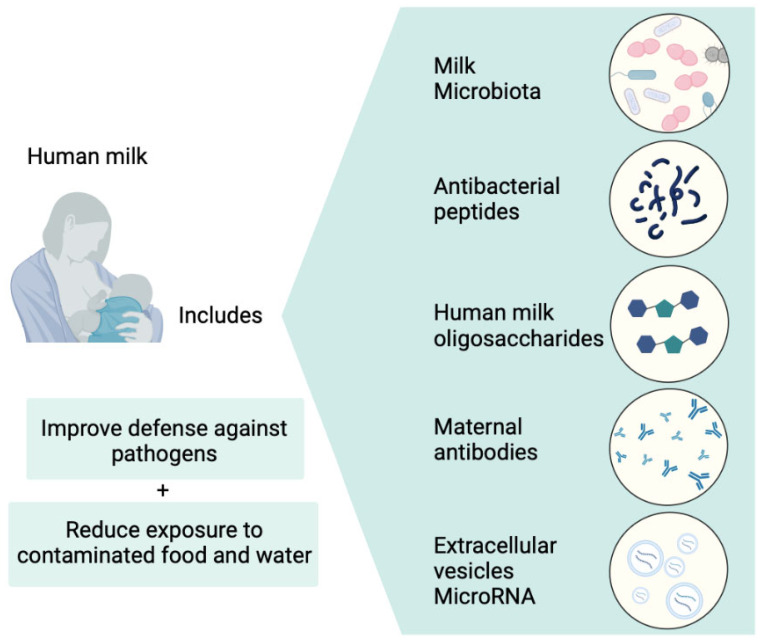
Components and functions of human breast milk.

Human breast milk serves as the primary source of energy for humans, playing a pivotal role in molding the early development of intestinal microorganisms. Additionally, it functions as a crucial reservoir of biologically valuable components, encompassing essential probiotic microorganisms and various other elements [12,13,14] (Figure 1). Various reports have established that human milk contains a significant amount of probiotic bacteria [15] estimated to be in the range of 10^1^–10^7^ colony-forming units (CFU) per mL [16]. According to Sakwinska et al., *Bifidobacterium lactis* NCC2818 was observed at a concentration of 5.5 × 10^2^ CFU/mL, and *Lactobacillus johnsonii* NCC533 was observed at a concentration of 2.4 × 10^2^ CFU/mL in human milk [16]. These various lactic acid bacteria (LAB), along with the diverse nutrients found in human milk, constitute the primary components of the early human gut microbiota. Generally, numerous strains of LAB can produce bacteriocin or antibacterial proteins that were proven effective to *S. aureus*, *Pseudomonas fluorescens*, *Pseudomonas aeruginosa*, *Salmonella* Typhi, *Shigella flexneri*, *Listeria monocytogenes*, *Escherichia coli* O157:H7, and *Clostridium botulinum* [17]. Previous research has demonstrated that *Enterococcus faecalis* exhibits antimicrobial activity against pathogenic bacteria, including *Salmonella enterica* and *Pseudomonas aeruginosa* [18]. The potent antibacterial effect of *Enterococcus* sp. is primarily attributed to bacteriocins, which is similar to other lactic acid bacteria [19]. Bacteriocins emerge as a secure alternative to antibiotics since they undergo decomposition by proteolytic enzymes within the body, thereby mitigating the risk of fostering antibiotic resistance [20].

Hence, our research is conducted to isolate LAB from human breast milk, characterize the antibacterial substances being produced, and identify alternative antibiotic agents capable of effectively controlling MRSA while mitigating the emergence of antibiotic resistance side effects.

## 2. Materials and Methods

### 2.1. Isolation and Identification of Bacteria

The human milk was collected and approved by the Institutional Review Board of Gyeonggi University (KGU-20191018-HR-046-04). The human milk then experienced sequential dilution in 0.85% NaCl solution, leading to concentrations ranging from 10^−6^ to 10^−9^. Following that, the diluted solution was spread to bromocresol purple (BCP) agar (Eiken Chemical Co., Ltd., Tokyo, Japan) and incubated at 30 °C for 24 h. We used the property of BCP to turn yellow at low pH to select bacteria that formed a yellow ring around the colony and confirmed them as lactic acid bacteria. Subsequently, these colonies were transferred to De Man, Rogosa, and Sharpe (MRS) broth and agar (Difco, Detroit, MI, USA) for standard culture at 30 °C. To ascertain strain homology, the 16S rRNA gene sequence technique was deployed using primers 27F and 1492R. The resultant sequences were scrutinized via the EzBioCloud database (https://www.ezbiocloud.net/identify, accessed on 20 November 2023) as a tool for sequence analysis.

### 2.2. Bacterial Growth Conditions and Metabolite Preparation

To assess antimicrobial activity, we employed the indicator strains *Staphylococcus aureus* KCTC 3881 and *Staphylococcus aureus* CCARM 3089. *S. aureus* KCTC 3881 was sourced from the Korean Collection for Type Cultures (KCTC), and *S. aureus* CCARM 3089 was obtained from the Culture Collection of Antimicrobial Resistant Microbes (CCARM). Nutrient broth (NB; Difco, Detroit, MI, USA) was used under aerobic conditions at 37 °C for *S. aureus* KCTC 3881 and *S. aureus* CCARM 3089 as indicators. The selected LAB for the study was cultured in MRS broth at 37 °C for 48 h to produce mass metabolites for experiments. Subsequently, centrifugation was performed at 3500 rpm for 15 min at 4 °C. The supernatants were filtered through a 0.22 μm membrane filter to obtain the cell-free supernatant (CFS). The CFS underwent liquid–liquid fractionation with ethyl acetate (1:1; *v*/*v*) to obtain the ethyl acetate fraction (EA). The concentrated fractions, obtained using a centrifugal evaporator (EYELA, Tokyo, Japan) at 45 °C, were utilized for further assessments.

### 2.3. Agar-Well Diffusion Assay

LAB strains possessing antimicrobial properties were detected through an agar-well diffusion method [21]. Specifically, 20, 50, and 100 μL aliquots of CFS and EA fractions were dispensed onto 8 mm well on Mueller–Hinton Agar (MHA; Difco, Detroit, MI, USA) plates previously coated with indicator strains (1 × 10^6^ UFC/mL). The plates were then incubated at 37 °C for 24 h, and the diameter of the inhibitory zone was measured to evaluate the antibacterial activity of the LAB. To analyze the antimicrobial effects of LAB, the diameter of the inhibitory zone was measured in millimeters using a caliper tool.

### 2.4. Broth Microdilution Assay

The investigation utilized the broth microdilution method, in accordance with the guidelines set forth by the Clinical and Laboratory Standards Institute, to determine the minimum inhibitory concentration (MIC) of the specific LAB chosen for the study. The CFS of the isolated LAB underwent serial dilution and were then inoculated into a 96-well microtiter plate (Thermo Fisher Scientific, Waltham, MA, USA) with 100 μL in each well. Subsequently, each well received 100 μL of indicator strains in NB broth (1 × 10^6^ CFU/mL) and was incubated at 37 °C for 24 h. Following incubation, the optical density (OD) was measured at 595 nm using a microplate reader (Molecular Devices, San Francisco, CA, USA) to determine the minimum inhibitory concentration (MIC). MRS broth was utilized as a control.

### 2.5. Crystal Violet Assay for Biofilm Formation

To assess the impact of isolated LAB on biofilm formation, the CFS of LAB two-fold serial dilution and was inoculated into a 96-well microtiter plate with 100 μL placed in each well. Subsequently, each well received an additional 100 μL of indicator strains in NB broth (1 × 10^6^ CFU/mL) and was then incubated for 24 h at 37 °C. Following incubation, the liquid was removed, and the wells were dried and rinsed twice with distilled water (DW). Subsequently, 100 μL of a 0.1% crystal violet solution was added to each well and left to stain for 15 min at room temperature. Afterward, the wells were washed twice with DW and allowed to air dry. The extent of biofilm formation inhibition was determined by liberating the bound crystal violet with 33% acetic acid and measuring the absorbance at 595 nm using a microplate reader. As part of the biofilm formation assessment, MRS broth was utilized as a control.

### 2.6. Characterization of Antimicrobial Substances

The characterization of antimicrobial substances of CFS was conducted using a modified method by Maria et al. [22]. The analysis comprised three distinct treatment groups to assess these substances. In the first treatment, the CFS of isolated lactic acid bacteria underwent pH adjustment to 6.5 for neutralization, which was achieved with 0.1 M NaOH. The second treatment involved pH adjustment to 6.5 followed by catalase treatment at 0.5 mg/mL at 30 °C for 1 h. The third treatment included pH adjustment to 6.5 followed by digestion at 55 °C for 1 h with 0.1 mg/mL proteinase K. Subsequently, proteinase K activity was deactivated at 65 °C for 15 min to investigate its effect on antibacterial substances in the CFS. MRS broth with identical treatments in each group served as controls. All samples were diluted to the minimum inhibitory concentration (MIC) in the broth microdilution method, and these experiments were conducted in triplicate with OD595 measured at 0, 6, 12, 18, and 24 h.

### 2.7. Sodium Dodecyl Sulfate (SDS)–Polyacrylamide Gel Electrophoresis (PAGE) Antibacterial Assay

The SDS-PAGE was employed for the assessment of antibacterial compounds. The ethyl acetate fraction of isolated LAB CFS was combined with sample buffer containing Tris-HCl (pH 6.8), SDS 8% (*w*/*v*), glycerol 40% (*v*/*v*), and 2-mercaptoethanol 8% (*v*/*v*) as a reducing agent, and the mixture was subjected to heat at 100 °C for 5 min. Subsequently, the prepared sample was loaded onto a 15% SDS-PAGE gel and electrophoresed under a constant voltage of 100 V. Each electrophoretic procedure was concluded upon the migration of the tracking dye to a point 1 cm above the sealing portion of the gel cast.

Post-electrophoresis, one gel was stained for proteins using crystal violet 0.001% (*w*/*v*) in 10% (*v*/*v*) methanol and 1.5% (*v*/*v*) acetic acid as described in a previous study method [23]. For the other gel, we utilized the overlaid soft agar assay to identify protein bands demonstrating antibacterial activity. This gel underwent three washes in 10% methanol (15 min each) at room temperature to eliminate SDS. Subsequently, as described by [24], a soft nutrient agar medium (25 mL) containing cells of pathogens (approximately 1 × 10^6^ CFU/mL) was overlaid to cover the entire gel. Clear zones indicative of antibacterial activity were observed after overnight incubation at 30 °C.

### 2.8. Scanning Electron Microscope (SEM)

The study utilized dilutions of 10^8^ CFU/mL of *S. aureus* KCTC 3881 and *S. aureus* CCARM 3089, which were exposed to the MIC and 2 × MIC of CFS at 37 °C for 24 h. After incubation, the bacterial cells were fixed using 2.5% glutaraldehyde in PBS for 2 h at 4 °C. Subsequently, the samples underwent post-fixation in a 1% osmium tetroxide solution (*w*/*v*) for 1 h at the same temperature. The fixed samples were then dehydrated using ethanol solutions with progressively higher concentrations (30%, 50%, 70%, 80%, 90%, and 100%). Following dehydration, the bacteria were dried for 24 h under a fume hood with 100 μL of hexamethyldisilazane (Sigma Aldrich, St. Louis, MO, USA). The dried cells were mounted on carbon tape attached to a stub and imaged using a SU8010 scanning electron microscope from Hitachi, Tokyo, Japan. This experiment aimed to visualize the effects of CFS on the morphology and structure of *S. aureus* KCTC 3881 and *S. aureus* CCARM 3089 cells using scanning electron microscopy.

### 2.9. Statistical Analysis

Statistical analyses, including two-way ANOVA, were performed using GraphPad Prism 9, which was developed by GraphPad Software Inc. in La Jolla, CA, USA. The data, collected in three separate replications, were presented as mean ± standard deviation. Significance levels of *p* < 0.001, *p* < 0.01, and *p* < 0.05 were applied to all statistical assessments.

## 3. Results

### 3.1. Antibacterial Effect of Isolated LAB

**Figure 2:**
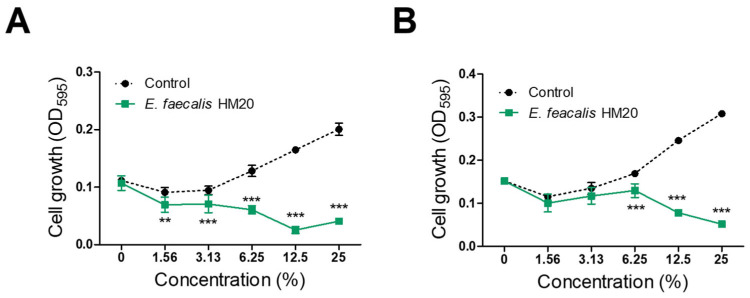
Antibacterial activity by CFS of *Enterococcus faecalis* HM20 against *Staphylococcus aureus* KCTC 3881 (**A**) and *Staphylococcus aureus* CCARM 3089 (**B**). Each experiment was repeated at least three times, and the findings are presented as the average ± standard deviation. The observed statistical significance *** *p* < 0.001 and ** *p* < 0.01 are in relation to MRS control group.

Twenty-six LAB strains were isolated from human milk utilizing the BCP plate pH color-changing characteristic. Among the identified strains, strain HM20 exhibited notable antibacterial activity against *S. aureus* following MIC value screenings. The findings indicated that HM20 CFS inhibited the growth of *S. aureus* KCTC 3881 and *S. aureus* CCARM 3089, featuring MIC values of 6.25% and 12.5% CFS, respectively (Figure 2). The analysis of the 16S rRNA gene phylogeny for strain HM20 disclosed its closest relative type strain, *Enterococcus faecalis* ATCC 19433^T^. Consequently, the strain was named *Enterococcus faecalis* HM20. MRS was diluted to the same concentration (1.56%, 3.13%, 6.25%, 12.5% and 25%) as the culture medium and used as the control group.

### 3.2. Inhibition of Biofilm Formation by E. faecalis HM20

**Figure 3:**
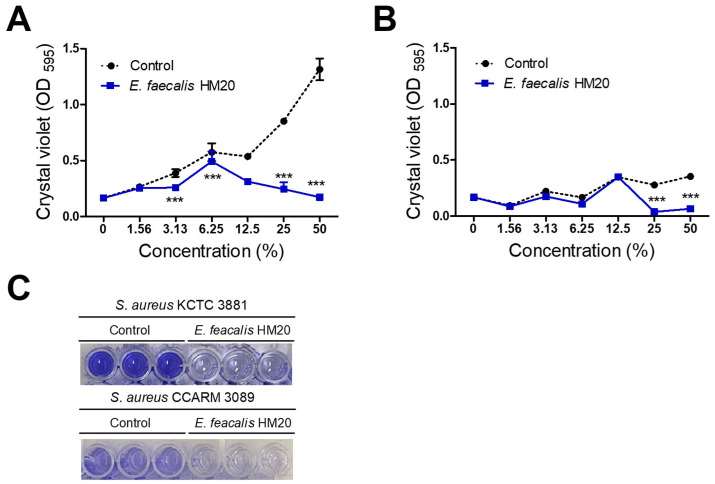
Antibiofilm formation activities of *Enterococcus faecalis* HM20 against *Staphylococcus aureus* KCTC 3881 (**A**) and *Staphylococcus aureus* CCARM 3089 (**B**). (**C**) Representative images of a 96-well plate showcasing the CV-stained biofilm. Each experiment was repeated at least three times, and the findings are presented as the average ± standard deviation. The observed statistical significance *** *p* < 0.001 is in relation to the control group.

This assessment presents the outcomes of the inhibitory impact on biofilm formation concerning skin pathogens, including *S. aureus* KCTC 3881 and *S. aureus* CCARM 3089 (MRSA). The MRS broth used in the control group showed a concentration-dependent increase in biofilm formation for both *S. aureus* KCTC 3881 and *S. aureus* CCARM 3089. In contrast, the CFS treatment group with HM20 exhibited a concentration-dependent growth increase at low concentrations, transitioning to a decline in biofilm formation at concentrations beyond a certain threshold for the indicator strains. Below these specific concentrations, the CFS of the HM20 treatment group exhibited no significant influence on the biofilm formation of *S. aureus* CCARM 3089. This reduction began at the 2 × MIC CFS concentration, which is 25% for *S. aureus* CCARM 3089 (MRSA), and the 1/2 × MIC CFS concentration, which is 3.13% for *S. aureus* KCTC 3881. Below these specific concentrations, the CFS of the HM20 treatment group exhibited no significant influence on the biofilm formation of *S. aureus* CCARM 3089 (Figure 3).

### 3.3. Characterization of Antimicrobial Substances of HM20

**Figure 4:**
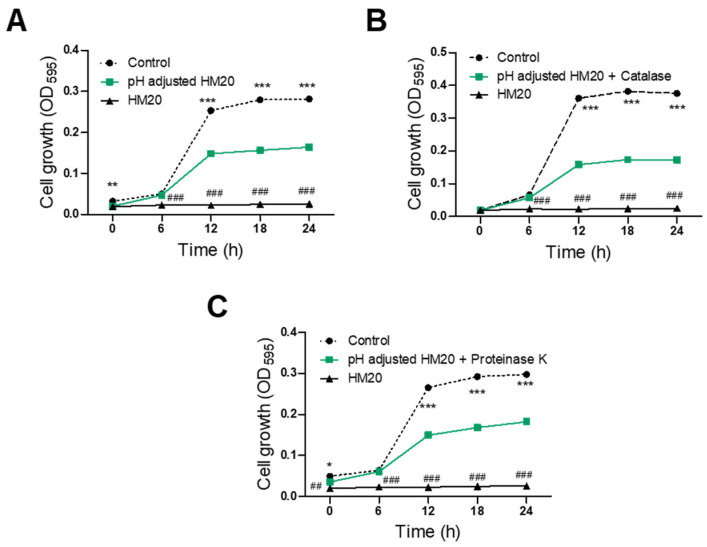
Antibacterial activity of *Enterococcus faecalis* HM20 on *Staphylococcus aureus* KCTC 3881 after treatment with pH adjustment and enzymes. (**A**) pH-adjusted; (**B**) pH-adjusted followed by catalase treatment; (**C**) pH-adjusted followed by proteinase K treatment. Each experiment was repeated at least three times, and the findings are presented as the average ± standard deviation. The observed statistical significance *** *p* < 0.001, ** *p* < 0.01 and * *p* < 0.05 vs. the same treatment control group. ### *p* < 0.001 and ## *p* < 0.01 vs. the MIC of HM20 treatment group.

The antibacterial activity of *E. faecalis* HM20 against *S. aureus* KCTC 3881 and *S. aureus* CCARM 3089 was examined following treatment with pH adjustment and various enzymes, including catalase and proteinase K. All three treatment groups (pH-adjusted, catalase-treated/pH-adjusted, and proteinase K-treated/pH-adjusted) showed a significant reduction in antimicrobial activity against *S. aureus* KCTC 3881 after 6 h compared to the untreated CFS of the HM20 group (Figure 4). Furthermore, the growth of *S. aureus* KCTC 3881 after 24 h of cultivation was significantly inhibited by the pH-adjusted CFS of the HM20 group with 42.15% reduction compared to the control group with 100% initial growth. The catalase-treated and proteinase K-treated CFS showed even greater *S. aureus* cell growth with 56.79% and 41.00%, respectively. In contrast, the untreated HM20 CFS exhibited a nearly complete inhibition of growth of 99.05%.

**Figure 5:**
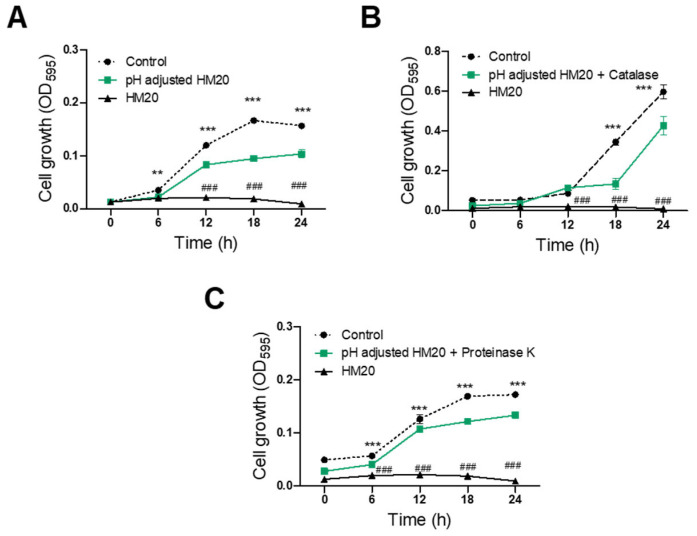
Antibacterial activity of *Enterococcus faecalis* HM20 on *Staphylococcus aureus* CCARM 3089 (MRSA) after treatment with pH adjustment and enzymes. (**A**) pH-adjusted; (**B**) pH-adjusted followed by catalase treatment; (**C**) pH-adjusted followed by proteinase K treatment. Each experiment was repeated at least three times, and the findings are presented as the average ± standard deviation. The observed statistical significance *** *p* < 0.001 and ** *p* < 0.01 vs. the same treatment control group. ### *p* < 0.001 vs. the MIC of HM20 treatment group.

Treating samples with *S. aureus* CCARM 3089 revealed significant antimicrobial effects from 6 h onwards for both pH-adjusted and proteinase-treated/pH-adjusted CFS compared to the control group (Figure 5). However, when catalase-treated/pH-adjusted CFS was used, a significant decline of antimicrobial effect was observed beginning from 18 h, resulting in a more gradual inhibition of growth. After 24 h of sample treatment, the pH-adjusted CFS of HM20 showed a 26.49% growth inhibition against *S. aureus* CCARM 3089 compared to the control group. Treatment with catalase or proteinase fluctuated the inhibition to 29.63% and 14.15%, respectively. On the other hand, the untreated HM20 CFS showed a complete inhibition of growth of 100.41%.

The growth inhibition results of the aforementioned pH adjustment group indicate an antimicrobial effect excluding the influence of organic acids in the sample. Treating the sample with catalase and adjusting the pH showed that the antimicrobial effect is from both organic acids and hydrogen peroxide. On the other hand, treating the sample with proteinase K suggested that peptides, not organic acids or bacteriocins, play a role in inhibiting growth. Based on these results, the antibacterial effect against *S. aureus* KCTC 3881 and *S. aureus* CCARM 3089 appears to involve a complex interplay of organic acids, hydrogen peroxide, and peptides, including bacteriocins.

### 3.4. HM20 Antibacterial Effect against MRSA on Agar-Well Diffusion after Purification

**Figure 6:**
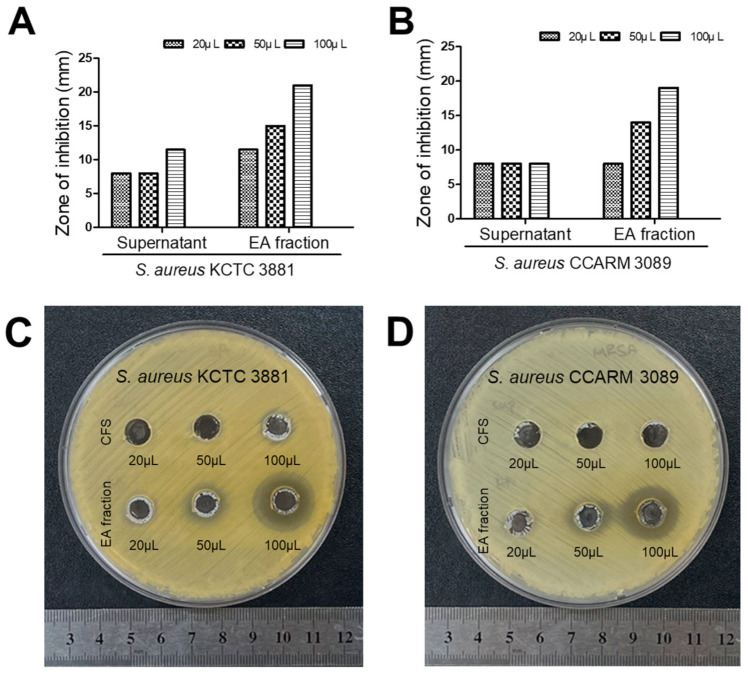
Antibacterial effect of HM20 cell-free supernatant and ethyl acetate fraction on agar-well diffusion. (**A**) Zone of inhibition bar graph of supernatant and ethyl acetate fraction against *Staphylococcus aureus* KCTC 3881; (**B**) zone of inhibition bar graph of supernatant and ethyl acetate fraction against *Staphylococcus aureus* CCARM 3089 (MRSA); (**C**) agar-well diffusion result of supernatant and ethyl acetate fraction against *Staphylococcus aureus* KCTC 3881; (**D**) agar-well diffusion result of supernatant and ethyl acetate fraction against *Staphylococcus aureus* CCARM 3089 (MRSA).

Based on the antibacterial activity test results for the enzyme-treated test, it was established that *E. faecalis* HM20 produces antimicrobial peptide. To purify this active substance, EA fractionation was executed, and their antibacterial effects were assessed in comparison to those of the CFS (Figure 6). Comparative results from the agar-well diffusion test showed that when 100 mg/mL of EA fraction was applied in 100 µL, inhibition zones of 21 mm and 19 mm were observed against *S. aureus* KCTC 3881 and *S. aureus CCARM* 3089, respectively. In contrast, the inhibition zone observed with 100 µL of CFS was 11.5 mm against *S. aureus* KCTC 3881 with no detected inhibition zone against *S. aureus* CCARM 3089.

### 3.5. Analysis of Active Bacteriocins in E. faecalis HM20 Using SDS-PAGE and Overlaid Agar

**Figure 7:**
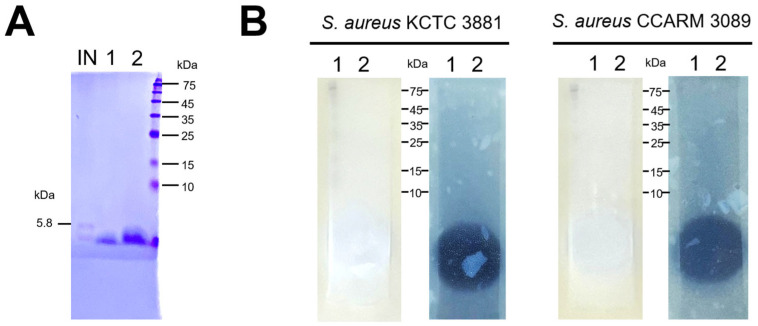
SDS-PAGE analysis (**A**) and overlaid agar assay (**B**) of *Enterococcus faecalis* HM20 ethyl acetate fraction. (IN) Insulin as a low-molecular-weight protein marker (50 μg); (1) ethyl acetate fraction (50 μg); (2) ethyl acetate fraction (100 μg).

The protein in the sample band (Figure 7A) was estimated to have a molecular weight of under 5000 Da when compared with standard proteins and insulin as a low-molecular-weight protein marker. It was confirmed that both bands exhibited activity against *S. aureus* KCTC 3881 and *S. aureus* CCARM 3089 in the bioassay. The EA fraction against *S. aureus* KCTC 3881 illustrated a larger inhibition zone compared to *S. aureus* CCARM 3089 (Figure 7B).

### 3.6. SEM Result of Pathogens with E. faecalis HM20 Treatment

**Figure 8:**
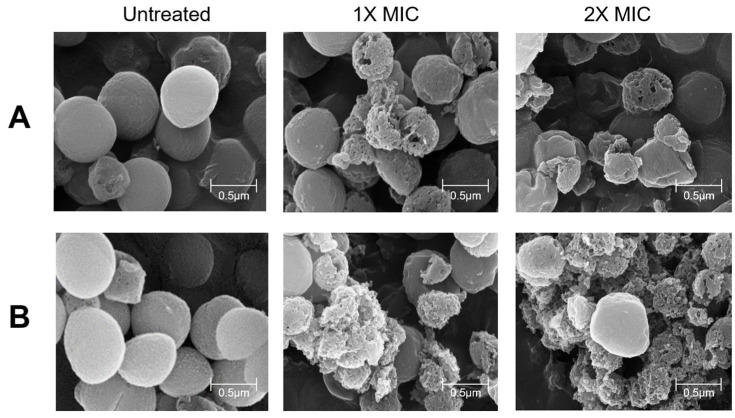
Scanning electron microscopy images (magnification: 100,000×, scale bar: 0.5 µm) confirming the antimicrobial effects of *Enterococcus faecalis* HM20 CFS on *Staphylococcus aureus* KCTC 3881 (**A**) and *Staphylococcus aureus* CCARM 3089 (**B**).

Changes in the cell surface induced by HM20 MIC and 2 × MIC were investigated using scanning electron microscopy, as illustrated in Figure 8. In the absence of treatment, *S. aureus* and MRSA cells exhibited a uniform spherical shape with an intact cell wall, presenting as grape-like clusters. Following exposure to HM20 CFS for 24 h, noticeable alterations in the shape of pathogen cells were observed, which were characterized by distortions, wrinkles, and gullies on the cell surface (Figure 8). HM20 CFS treatment resulted in some cells being ruptured, displaying visible cracks and pores, with a clear loss of integrity in the cell membrane and leakage of cellular constituents. Notably, the 2 × MIC HM20 CFS treatment led to a more significant disruption in cell morphology, presenting as shrunken spheres. The CFS of HM20 treatment caused severe damage, including the collapse of the cytoskeleton, disintegration of the cell membrane, and damage to the cell and cellular fragments in the background of the SEM image.

## 4. Discussion

In this study, we conducted research to suppress the emergence of antibiotic resistance and to discover safer antimicrobial substances against skin pathogens. Our study aimed to identify and analyze the characteristics of antimicrobial substances derived from human milk lactic acid bacteria (LAB), specifically focusing on their effectiveness against MRSA, which is a notorious multidrug-resistant pathogen responsible for various bacterial skin infections. The escalating prevalence of antibiotic-resistant pathogens underscores the urgency of exploring alternative treatments devoid of severe side effects or toxicity [25]. Consequently, there is a growing interest in research exploring the potential application of LAB metabolites in inhibiting MRSA infection.

Previous studies on *Enterococcus faecalis* have reported its anti-hair loss properties [26], as well as its potential for improving atopic dermatitis [27], providing anti-inflammatory effects [28], and exhibiting antimicrobial activity [18]. However, there is a paucity of research on the control of MRSA using *E. faecalis* or its metabolite. Significantly, the CFS of *Enterococcus faecalis* HM20 demonstrated substantial reductions in the growth and biofilm formation of the MRSA (Figure 2 and Figure 3), complementing existing reports on LAB antibiofilm effects [17,29].

The *Staphylococcus aureus* KCTC 3881 was recorded to produce stronger biofilm forming compared to MRSA in Figure 3. These characteristics appear to result from a mechanism distinct from non-specific antibiotic-resistant strains that fortify their biofilm to prevent antibiotic penetration [30]. In the case of methicillin-resistant strains, the observed traits are attributed to the production of enzymes that degrade the action of antibiotics, rendering them ineffective [31]. Despite the differences in biofilm formation between both pathogens, our study confirms that the CFS of HM20 effectively inhibits biofilm formation in both strains at concentrations of 25% CFS or higher. However, no antibacterial effect of HM20 strain supernatant against MRSA was observed in the agar-well diffusion test. The inconsistency between MIC and agar-well diffusion methods can be due to their qualitative versus quantitative nature, differences in diffusion characteristics, and the diverse composition of LAB supernatant. Additionally, the differences can be associated with variations of the opaque material in the broth which can affect the OD of the sample; this was noted congruently with the findings of Bubonja-Šonje et al. [32].

The analysis of antimicrobial effects using pH-adjusted and enzyme-treated CFS of HM20 successfully identified organic acids, hydrogen peroxide, and peptides as key contributors to its antimicrobial activity. However, the individual antimicrobial effects of these substances did not fully match the potency of the untreated CFS, suggesting a potential synergistic interaction among them. This finding aligns with previous studies and implies that the combined action of organic acids and hydrogen peroxide might weaken the bacterial cell wall, enhancing the penetration and efficacy of peptides, such as bacteriocins [33,34].

Through the investigation of two-dimensional gel electrophoresis and subsequent agar-overlay assays, the objective was to characterize a peptide effective against MRSA. This trait, coupled with an isoelectric point of approximately 5 kDa in molecular weight, classifies it as a peptide with potent activity against two chosen pathogens related to skin diseases. In this experimental process, despite exposure to high temperatures, the sample maintained a stable antimicrobial effect and exhibited a size below 5 kDa. These characteristics are reminiscent of antimicrobial peptides known as Enterocins produced by *Enterococcus* sp., which are stable at high temperatures and typically range in size from 4.5 to 4.7 kDa [35]. This suggests that the antimicrobial effect of HM20 may be derived from enterocin.

A recent study observed that antibiotic-resistant strains exhibit increased cell wall breaking as a result of exposure to antibiotics such as erythromycin and clarithromycin. This phenomenon leads to cell shrinkage and loss of viability [36]. In our research, the treated samples showed *Staphylococcus aureus* cell shrinkage and cell wall breaking (Figure 8), possibly indicating the CFS of HM20 ability to inhibit protein synthesis.

Furthermore, enterocin is known to inhibit the growth of target indicator strains by methods such as disrupting the peptidoglycan layer of the cell wall and forming pores in the cell membrane [37]. With this information, we can more robustly support the hypothesis that the antimicrobial substance of HM20 is derived from the production of enterocin.

Considering the analysis of antimicrobial activity from strain HM20 in enzyme-treated samples, organic acids, hydrogen peroxide, or bacteriocins are commonly implicated. Therefore, to utilize strain HM20 as a material against MRSA, additional analysis of metabolites produced by HM20, apart from the antimicrobial peptides under 5 kDa elucidated in this study, is necessary. Moreover, additional research evaluating clinical safety and formulation stability appears to be required.

## 5. Conclusions

In this study, our aim was to develop a safe and effective antibacterial material without the side effects associated with antibiotic-resistant strains. Notably, the human milk-derived strain *E. faecalis* HM20 exhibited potent antimicrobial activity against skin pathogens, including MRSA. This activity was attributed to the presence of organic acids, hydrogen peroxide, and bacteriocins. The isolated HM20 peptide demonstrated properties similar to enterocin. Further research is needed on the identification of the antimicrobial substance in HM20, its human safety, and its antimicrobial activity stability under various conditions. This could position HM20 as a promising candidate for a new antibacterial material to combat skin pathogens, highlighting the potential for innovative antibacterial solutions.

## Data Availability

Data can be made available upon request.
